# Supplementary figures and images for: Diffusion and distal linkages govern interchromosomal dynamics during meiotic prophase
Source: Proc Natl Acad Sci U S A. 2022 Mar 18;119(12):e2115883119. doi: 10.1073/pnas.2115883119 (PMC8944930; doi:10.1073/pnas.2115883119)

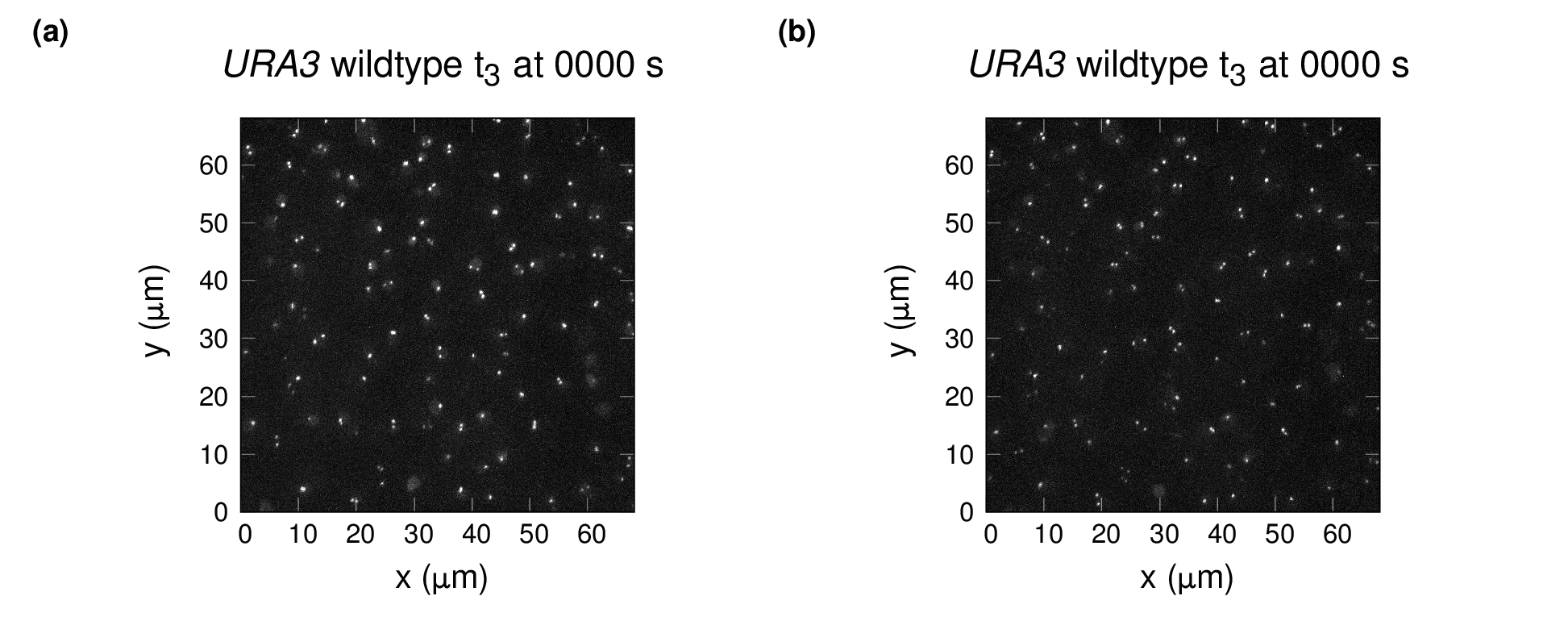

Supplement: Supplementary File [file pnas.2115883119.sm01.gif]

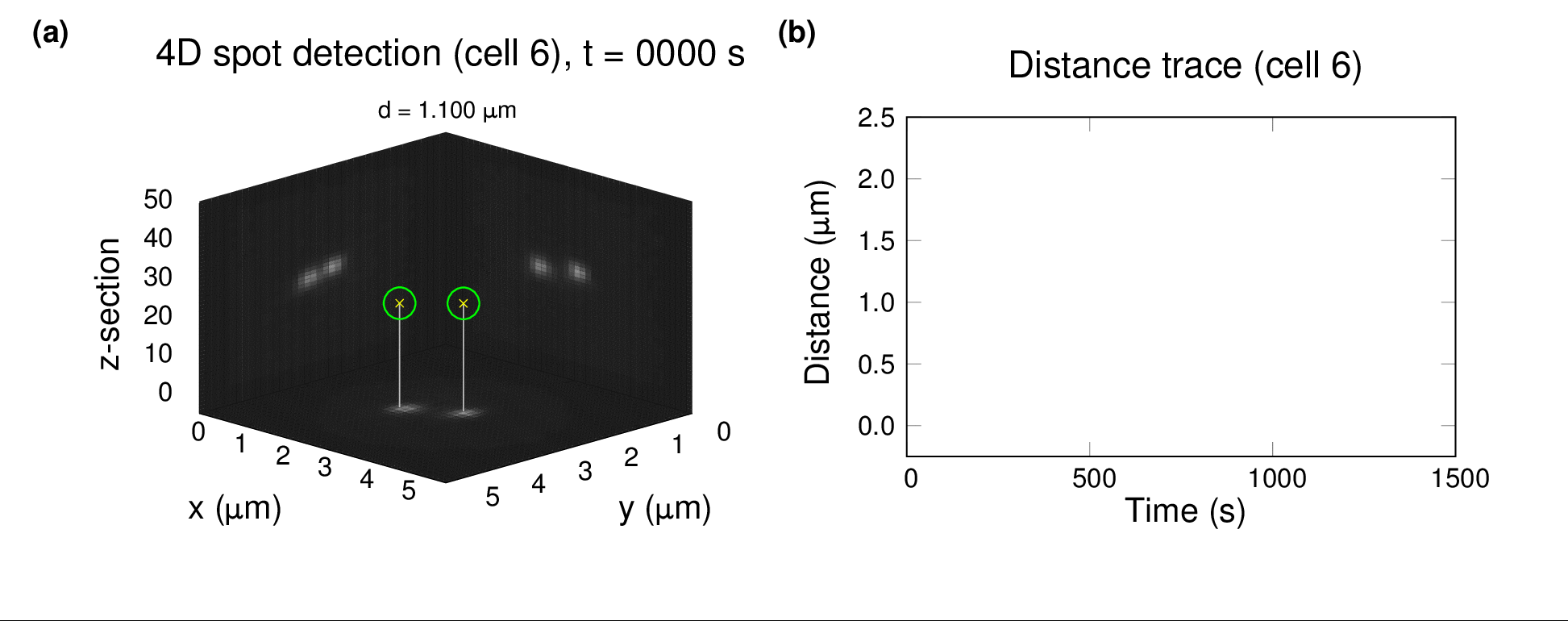

Supplement: Supplementary File [file pnas.2115883119.sm02.gif]

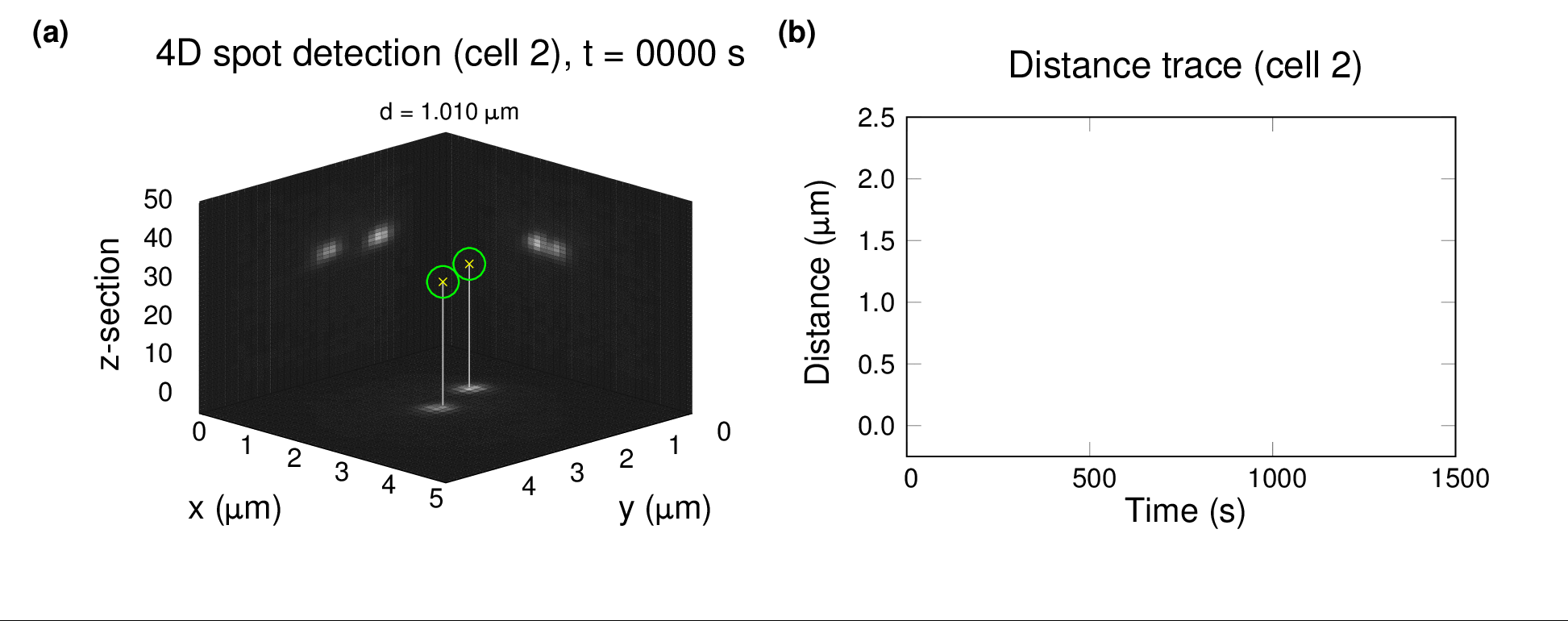

Supplement: Supplementary File [file pnas.2115883119.sm03.gif]

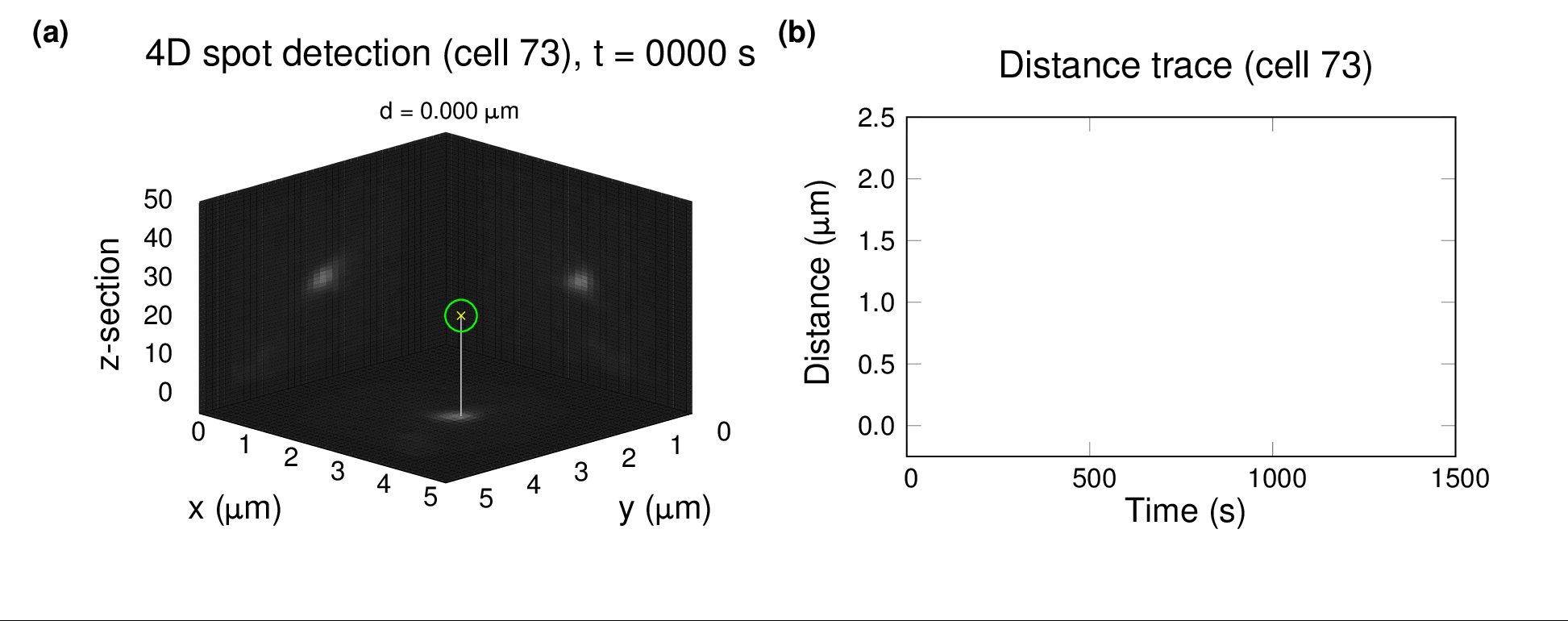

Supplement: Supplementary File [file pnas.2115883119.sm04.gif]
